# Characterization and Prediction of Epoxy Resin Curing Behavior Using a Data-Driven Kinetic Model Based on Dual Kriging

**DOI:** 10.3390/polym18212643

**Published:** 2026-10-25

**Authors:** Chao Yang, Jinxin Huang, Xiaolong Xing, Yueming Zhou, Bin Yang, Aiqing Ni, Jihui Wang

**Affiliations:** 1 School of Materials Science and Engineering, Wuhan University of Technology, Wuhan 430070, China; 2 Xi’an Aerospace Composites Research Institute, Xi’an 710025, China; 3 State Key Laboratory of Advanced Technology for Material Synthesis and Processing, Wuhan University of Technology, Wuhan 430070, China

**Keywords:** cure behavior, Dual Kriging, differential scanning calorimetry (DSC), fiber Bragg grating (FBG), finite element (FE)

## Abstract

A data-driven kinetic framework based on Dual Kriging is developed to represent epoxy curing behavior and supply a local cure-rate function for finite element (FE) analysis. Error-controlled compression reduces 7200 isothermal observations to 187 points, with maximum and mean normalized reconstruction errors of 0.45% and 0.069%, respectively. The compressed interpolant reproduces the E-51 conversion histories measured by differential scanning calorimetry (DSC), including their late-stage plateaus. A separable 245-node rate representation is implemented in a sequential thermo-chemo-mechanical analysis, combining E-51 experimental data at 55–70 °C with auxiliary Kamal–Sourour data at 30–50 °C. The two kinetics models produce similar temperature and strain histories during staged curing; both underestimate the measured temperature peak and late-stage contraction. Temperature interpolation is evaluated separately using independent DSC measurements for another epoxy system, with the rate model constructed entirely from experimental data. With the complete 70 °C curve withheld and six temperatures used for training, the peak-normalized rate root mean square error (RMSE) is 1.11%, and the apparent-conversion RMSE is 0.00271. Withholding each of the five interior temperatures in turn gives rate errors of 1.11–5.58%. Within this dataset, expanding the training-temperature set improves prediction more than increasing the node count along existing curves.

## 1. Introduction

The curing of thermosetting epoxy resins couples chemical reaction, heat transfer, and mechanical-property development. Reaction heat changes the local temperature, while temperature controls the rate of cure; thermal deformation and chemical shrinkage subsequently contribute to residual stress [1,2]. Process models therefore combine a thermal–chemical calculation with a constitutive description of the evolving resin [3,4,5]. Within this framework, cure kinetics determines the heat-source history and the degree of cure supplied to the mechanical analysis. An adequate kinetics description is consequently important for predicting the temperature and strain response of resin systems subjected to staged curing schedules.

Differential scanning calorimetry (DSC) provides an experimental basis for identifying cure kinetics. Phenomenological equations, including the Kamal–Sourour autocatalytic model, express the reaction rate through a small set of temperature-dependent parameters and are convenient for numerical implementation [6]. Early studies established kinetic descriptions for epoxy systems through phenomenological formulations and experimental parameter identification [7,8]. Extensions incorporating diffusion effects have been introduced to describe the reaction slowdown at high conversion, where reduced molecular mobility and vitrification become significant [9,10]. Nevertheless, the predictive capability of these models depends on both the assumed functional form and the experimental conditions used for parameter identification. Accurately representing late-stage reaction behavior remains challenging in isothermal processing, particularly during prolonged dwell periods [9].

Approaches that reduce reliance on a prescribed reaction equation offer an alternative. Tao et al. [11] incorporated model-free kinetics into finite element curing calculations through Abaqus user subroutines. More recently, data-driven methods have been investigated at different levels of the curing problem. Hui et al. [12] trained a neural-network kinetics model using non-isothermal DSC data, included the temperature rate among its inputs, and coupled the model to a thermochemical analysis. Wu et al. [13] used numerical simulation data to develop predictors of curing curves. These studies demonstrate the potential of data-driven approaches for representing curing behavior. Predicting a complete curing trajectory under a specified thermal schedule, however, differs from constructing the local kinetic relation required by a finite element solver. In coupled thermo-chemo-mechanical analysis, the cure rate must be evaluated repeatedly at each integration point according to the instantaneous degree of cure and temperature. A suitable data-driven kinetic representation should therefore preserve the measured local curing behavior while remaining suitable for numerical integration.

Recent work has further broadened the available approaches to kinetic identification. Wu et al. [14] compared autocatalytic, neural-network, and angle information-enhanced radial-basis-function models, while Kim et al. [15] introduced an ensemble cure kinetics network. These developments motivate the investigation of data-driven rate descriptions alongside conventional equations. Their practical value depends on the available experimental coverage and on how interpolation errors propagate during numerical integration. Accurate reconstruction of measured DSC curves alone does not establish predictive accuracy at unmeasured temperatures. For a resin characterized at a limited number of isothermal temperatures, a useful objective is to construct a compact local rate representation, evaluate its temperature interpolation capability, and examine its effect on the simulated curing response.

Dual Kriging provides a suitable framework for investigating this objective. Based on intrinsic random-function theory, it combines a drift function describing the overall trend with a correction constructed from sampled observations [16,17]. Kriging is closely related to Gaussian process regression, and their prediction equations can coincide under compatible mean, covariance, and observation assumptions [18]. Here, Dual Kriging is used as a deterministic interpolant with polynomial constraints and a generalized covariance, focusing on curve reconstruction and local rate evaluation without estimating posterior uncertainty. This formulation is selected because it accommodates error-controlled data compression followed by interpolation of the retained nodes and provides a local rate function whose coefficients can be determined in advance for repeated evaluation during finite element analysis. Applied to cure kinetics, Dual Kriging can represent the reaction rate as a function of degree of cure and temperature without prescribing an autocatalytic reaction equation. Its accuracy and computational efficiency depend on the sampling density, drift order, and generalized covariance. Closely spaced samples can increase the size of the interpolation system while providing limited additional information, whereas excessive compression may remove features needed to reproduce the curing response. Furthermore, local rate errors can accumulate during time integration and affect the predicted temperature and strain histories. Data reduction, interpolation accuracy, and the resulting curing response are therefore considered together to establish a compact kinetic representation that preserves the measured behavior and is suitable for finite element analysis.

In this study, Dual Kriging is used to represent the isothermal curing behavior of E-51 epoxy resin and construct a local rate relation, dα/dt=f(α,T), where α is the degree of cure and T is the local resin temperature. Isothermal DSC measurements at 55, 60, 65, and 70 °C provide the experimental data. Error-controlled compression reduces the degree-of-cure dataset, and leave-one-out cross-validation is used to compare candidate drift–covariance combinations. Because the available E-51 measurements do not cover the low-temperature stage of the curing schedule, auxiliary data generated using a diffusion-corrected Kamal–Sourour model are used to supplement this temperature range in the present case study. The local rate function is implemented in Abaqus within a sequential thermo-chemo-mechanical analysis to predict temperature and strain during staged curing. The Kamal–Sourour model serves as a reference for comparisons of the isothermal curing curves and finite element predictions, while embedded fiber Bragg grating (FBG) sensors provide experimental temperature and strain histories. An independent DSC dataset for another epoxy system is also used to evaluate temperature interpolation by withholding complete isothermal curves, with the rate model constructed solely from experimental data. The contribution of this work lies in combining error-controlled DSC-data compression with a separable local rate representation and evaluating both reconstruction accuracy and prediction at withheld temperatures. The FE application further examines how the kinetics description affects temperature and strain during staged curing.

## 2. Materials and Experimental Methods

### 2.1. Materials and Specimen Preparation

Epoxy resin E-51 and an amine curing agent were supplied by the China Academy of Engineering Physics (Mianyang, China). The resin and curing agent were mixed at a mass ratio of 100:25 at room temperature. The mixture was used for DSC characterization and for the resin specimen monitored during staged curing.

### 2.2. DSC Measurements

A differential scanning calorimeter (DSC 2500) from TA Instruments (New Castle, DE, USA) was used to characterize cure kinetics under ramp and isothermal conditions. A quantity of 5–10 mg of resin was poured into a sealed aluminum pan and stored in a freezer before each test. The dynamic measurements were performed in a ramp mode at 10, 15, and 20 °C/min. The temperature ranged between −80 °C and 245 °C. Isothermal DSC tests were conducted at 55, 60, 65, and 70 °C, each lasting 360 min. The flow rate of the inert purge gas, which was nitrogen in this case, was 50 mL/min. Under both the ramp and isothermal conditions, the heat flow, temperature, and time were recorded. Baseline subtraction was applied in the data processing procedure.

### 2.3. FBG Monitoring and Curing Schedule

FBG sensors manufactured by Shangning Zhigan (Beijing) Technology Co., Ltd. (Beijing, China) were embedded in the E-51 epoxy resin to monitor temperature and axial strain during staged curing. The measured histories were used to evaluate the corresponding finite element predictions. The relative Bragg wavelength shift is expressed as [19]:

(1) $$\frac{\Delta \lambda_{B}}{\lambda_{B}}=S_{T}\Delta T+S_{\varepsilon }\Delta \varepsilon ,$$

where $\lambda_{B}$ is the Bragg center wavelength at the reference state, and $\Delta \lambda_{B}$ is the wavelength shift relative to that state. ΔT and Δε represent the changes in temperature and axial strain, respectively. $S_{T}$ and $S_{\varepsilon }$ are the normalized temperature and strain sensitivity coefficients, defined as $S_{T}=\frac{k_{T}}{\lambda_{B}}$ and $S_{\varepsilon }=\frac{k_{\varepsilon }}{\lambda_{B}}$, where $k_{T}$ = 9.8 pm/°C, $k_{\varepsilon }$=1.04 pm/με.

**Figure 1:**
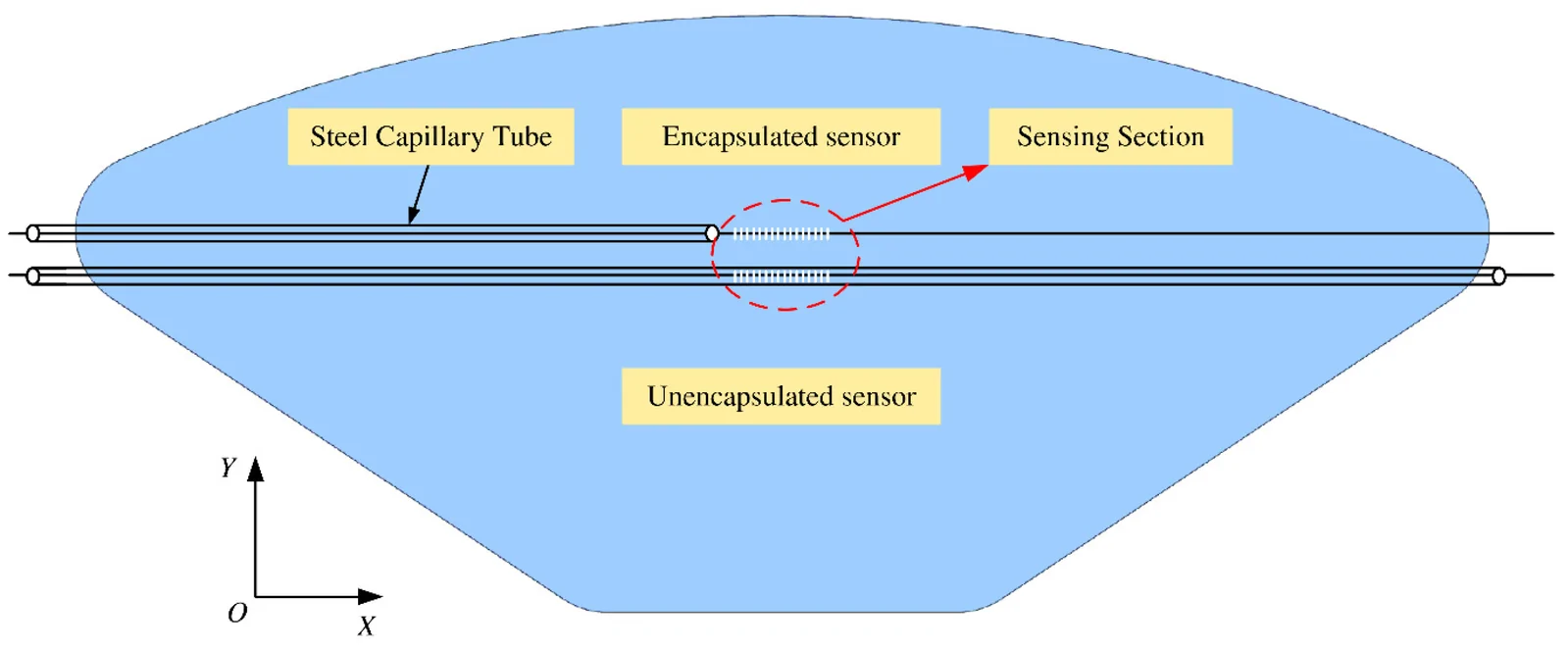
Arrangement of the encapsulated and unencapsulated FBG sensors for temperature and strain monitoring during resin curing.

As illustrated in Figure 1, an encapsulated FBG and an unencapsulated FBG were positioned within the resin. The temperature-reference FBG was encapsulated in a steel capillary tube to isolate its sensing region from resin deformation, allowing its wavelength shift to reflect temperature changes. The unencapsulated FBG responded to both temperature changes and axial strain transferred from the surrounding resin.

Assuming that the two sensors experienced the same local temperature, the temperature history was determined from the encapsulated FBG using its temperature sensitivity. The temperature-induced wavelength shift of the unencapsulated FBG was then calculated and subtracted from its measured wavelength shift. The remaining shift was divided by its strain sensitivity to obtain the axial strain history. All wavelength changes were referenced to the initial readings.

Two identical experiments were conducted under the same curing conditions. The prescribed curing schedule consisted of an initial hold at 30 °C for 24 h, followed by heating to 70 °C at 2 °C/min and holding at 70 °C for 3 h. The specimen was subsequently allowed to cool naturally to room temperature. This schedule describes the imposed thermal conditions; the local resin temperature was measured by the encapsulated FBG to capture the thermal response during curing.

A separate cooling characterization test was conducted to obtain the natural-cooling history. The encapsulation structure was heated to 60 °C, and wavelength measurements were recorded during natural cooling after the FBG wavelength had stabilized. The measured cooling data were fitted using a nonlinear function for use in the numerical analysis. The monitoring-point correspondence and implementation of the cooling boundary condition are described in Section 4.2.

## 3. Development of the Dual Kriging Cure Kinetics Model

A Dual Kriging framework is adopted to represent the isothermal DSC data and construct a local cure-rate function for finite element analysis. Data compression and cross-validation are used to evaluate the accuracy and computational cost of the degree-of-cure representation. The local rate relation, dα/dt=f(α,T), provides the kinetics input for the curing simulation described in Section 4. A diffusion-corrected Kamal–Sourour model is used as the reference kinetics description.

### 3.1. Data Preparation and Normalization

The degree of cure and cure rate are calculated by dividing the cumulative specific reaction heat and specific heat-flow rate, respectively, by a common reference reaction heat of 410.1 J/g. This value is the largest of the three dynamic DSC integrals reported for E-51 [20] and is also used to calculate the reaction heat source in the FE analysis.

The data are organized as $\left(t_{i},T_{i},\alpha_{i},r_{i}\right)$. Time, temperature, and degree of cure are normalized using their respective ranges, while the cure rate is scaled by its maximum value:

(2) $$z_{norm}=\frac{z-z_{min}}{z_{max}-z_{min}},\;z\in \{t,T,\alpha \},\;r_{norm}=\frac{r}{r_{max}}.$$

**Figure 2:**
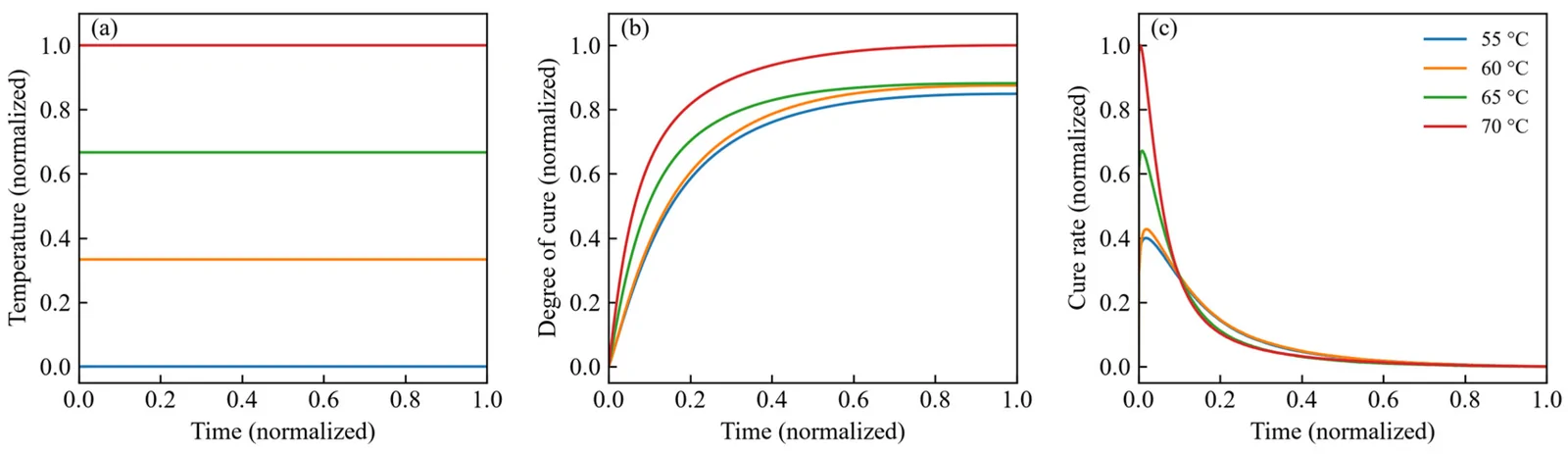
Normalization of isothermal measurements: (**a**) temperature; (**b**) degree of cure; (**c**) cure rate.

The extrema are determined over the combined isothermal dataset, and $r_{max}$ is its maximum measured cure rate. Figure 2 illustrates the normalized temperature, degree of cure, and cure rate.

The isothermal conversion histories are calculated over the recorded 360 min period without residual-heat compensation. The subsequent numerical scaling in Equation (2) places the interpolation variables on comparable scales.

For curve compression, normalized time and temperature are the interpolation inputs, and normalized degree of cure is the response. The input vector is therefore $x={\left(t_{norm},T_{norm}\right)}^{T}$. The normalization parameters are retained throughout compression, cross-validation, and interpolation evaluation.

### 3.2. Dual Kriging Formulation

In the Dual Kriging formulation, the response is decomposed into a polynomial drift and a residual component [16,17]:

(3) $$U\left(x\right)=a\left(x\right)+W\left(x\right),$$

where $a\left(x\right)$ describes the overall trend and $W\left(x\right)$ represents the variation about that trend. The interpolated response is expressed as

(4) $$\hat{u}(x)=p^{T}(x)c+\sum_{i=1}^{N}b_{i}K(∥x-x_{i}{∥}_{2}),$$

where $u=\alpha_{norm}$ for the degree-of-cure representation, N is the number of interpolation points, and $x_{i}$ is the normalized coordinate of the ith point. The vector p contains the polynomial basis functions, c contains the drift coefficients, and $b_{i}$ are the interpolation weights. The notation $∥\cdot {∥}_{2}$ denotes the Euclidean norm, and K is the radial kernel used in the generalized covariance representation.

Constant, linear, and quadratic drift functions are considered, with the corresponding polynomial bases

(5) $$\begin{array}{ll} p_{0}\left(x\right) & ={\left(1\right)}^{T},\\ p_{1}\left(x\right) & ={\left(1,x,y\right)}^{T},\\ p_{2}\left(x\right) & ={\left(1,x,y,x^{2},y^{2},xy\right)}^{T}, \end{array}$$

where $x=t_{norm}$ and $y=T_{norm}$.

The interpolation conditions and polynomial constraints lead to the augmented system

(6) $$\left[\begin{array}{cc} K & P\\ P^{T} & 0 \end{array}\right]\left[\begin{array}{c} b\\ c \end{array}\right]=\left[\begin{array}{c} u\\ 0 \end{array}\right],$$

where $K_{ij}=K\left(h_{ij}\right)$, $h_{ij}=∥x_{i}-x_{j}{∥}_{2}$, and $P_{ij}=p_{j}\left(x_{i}\right)$. The vector **u** contains the normalized responses at the interpolation points.

The candidate kernels are

(7) $$K_{lin}(h)=h,K_{cub}(h)=h^{3},K_{log}(h)=\left\{\begin{array}{l} h^{2}lnh,\;h>0,\\ 0,\;\;\;\;h=0. \end{array}\right.$$

The cubic and logarithmic kernels are paired with linear or quadratic drift functions. Distinct input points and a full-column-rank polynomial matrix are required. The coefficients are obtained by solving the augmented system, and numerical solvability is checked before comparing the candidate models.

### 3.3. Error-Controlled Data Compression

Data compression reduces the interpolation system size while preserving the measured isothermal degree-of-cure profiles. The initial point set contains the endpoints and local extrema of the assembled response sequence. A provisional interpolant is constructed using linear drift and a linear covariance kernel.

The local reconstruction error is defined as

(8) $$e_{i}=|{\hat{\alpha }}_{norm,i}-\alpha_{norm,i}|,$$

where ${\hat{\alpha }}_{norm,i}$ and $\alpha_{norm,i}$ are the interpolated and measured normalized degrees of cure, respectively.

Errors are evaluated at the original data points. Retained points and points with zero normalized response are excluded from the compression criterion. If the maximum error exceeds the prescribed tolerance, points corresponding to local maxima of the absolute error are added to the retained set. The interpolant is then updated, and the procedure is repeated until the tolerance is satisfied. A tolerance of 0.5% is used to obtain the compressed dataset for cross-validation.

Reconstruction errors are additionally evaluated over all original observations, including zero-response and retained points. The resulting maximum and mean normalized errors are 0.44762% and 0.069095%, respectively.

Compression performance is assessed using the retained point count, maximum and mean reconstruction errors, and percentage reduction in dataset size:

(9) $$\eta_{comp}=\left(1-\frac{N_{ret}}{N_{orig}}\right)\times 100\%,$$

where $N_{orig}$ and $N_{ret}$ are the original and retained point counts, respectively.

### 3.4. Model Evaluation and Local Cure-Rate Representation

Leave-one-out cross-validation is performed on the compressed isothermal dataset to compare the admissible drift–covariance combinations. Each retained degree-of-cure value is predicted using the remaining points. The local absolute error is defined as

(10) $$\delta_{j}=\left|{\hat{u}}^{\left(-j\right)}(x_{j})-u_{j}\right|,$$

where the superscript $\left(-j\right)$ denotes omission of the jth point. The retained point set is determined before cross-validation, and the normalization parameters remain unchanged throughout the procedure.

The cumulative, mean, and maximum absolute errors are calculated as

(11) $$E_{cum}=\sum_{j=1}^{N}\delta_{j},E_{MAE}=\frac{1}{N}\sum_{j=1}^{N}\delta_{j},E_{max}=\underset{j}{max}\delta_{j}.$$

The leave-one-out residuals are obtained algebraically from the augmented interpolation system and checked against explicit refitting at selected points. These metrics assess interpolation performance within the retained degree-of-cure dataset.

The LOOCV errors are conditional on the compressed point set. Prediction at unobserved temperatures is evaluated using complete-curve holdouts in Section 5.5.

Computational cost is assessed by evaluating each constructed interpolant at all original isothermal data points. After warm-up, the median CPU time per evaluation and its interquartile range are calculated from seven batches of repeated evaluations. Only interpolation evaluation is timed.

The local rate formulation uses degree of cure and temperature as its state variables. The local rate surface uses linear drift and cubic generalized covariance. Experimental rate data at 55, 60, 65, and 70 °C are expressed using the common reaction-heat reference defined in Section 3.1. Because DSC measurements are unavailable for the low-temperature stage of the E-51 curing schedule, auxiliary rate curves at 30, 40, and 50 °C are generated using the Kamal–Sourour reference model in Section 3.5. These temperatures include the initial 30 °C dwell and bridge the interval to the lowest experimental temperature. The E-51 implementation therefore combines experimental and model-generated rate data.

The rate dataset is represented at $N_{T}$ temperature nodes and $N_{\alpha }$ degree-of-cure nodes. Uniformly spaced degree-of-cure nodes are defined as

(12) $$\alpha_{i}=\frac{i-1}{N_{\alpha }-1},i=1,\ldots ,N_{\alpha }.$$

The experimental cure-rate curves were mapped onto these common degree-of-cure nodes, with extrapolation applied beyond the measured conversion range where necessary. At each temperature node, the rate is interpolated along the degree-of-cure coordinate using a cubic kernel and linear drift:

(13) $$S_{j}(\alpha )=c_{0j}+c_{1j}\alpha +\sum_{i=1}^{N_{\alpha }}b_{ij}|\alpha -\alpha_{i}{|}^{3},j=1,\ldots ,N_{T},$$

where $c_{0j}$ and $c_{1j}$ are the drift coefficients, and $b_{ij}$ are the kernel weights. Interpolation in the temperature direction uses the same kernel and drift, giving

(14) $$\frac{d\alpha }{dt}=f(\alpha ,T)=\sum_{j=1}^{N_{T}}L_{j}(\theta )S_{j}(\alpha ),\theta =\frac{T-T_{min}}{T_{max}-T_{min}},$$

where $T_{min}$ and $T_{max}$ are the temperature bounds of the combined rate dataset. The temperature interpolation functions $L_{j}$ satisfy $L_{j}(\theta_{k})=\delta_{jk}$, where $\theta_{k}$ is the normalized temperature of the kth node and $\delta_{jk}$ is the Kronecker delta. The coefficients in both interpolation directions are determined using the interpolation conditions and polynomial constraints described in Section 3.2. For the local rate representation, 35 uniformly spaced degree-of-cure nodes are prescribed at each of the seven temperatures–30, 40, 50, 55, 60, 65, and 70 °C–giving 245 rate nodes in total.

The resulting rate function is evaluated at the local degree of cure and temperature during the thermal–chemical analysis. The rate is used to update the degree of cure and calculate the reaction heat source.

The 187-point conversion interpolant is used to reconstruct the measured time–temperature histories, whereas the 245-node rate surface supplies the local kinetics for FE integration. Compression and computational-cost metrics reported for the conversion interpolant refer to that representation. The effect of experimental temperature coverage on local rate prediction is examined using the independent DSC dataset in Section 5.5.

### 3.5. Reference Kamal–Sourour Model

A diffusion-corrected Kamal–Sourour model [6], previously applied to the E-51 resin system [20], is used as the reference kinetics description:

(15) $$\frac{d\alpha }{dt}=\frac{\left(K_{1}+K_{2}\alpha^{m}\right){\left(1-\alpha \right)}^{n}}{1+e^{D\left(\alpha -\alpha_{c}\right)}},$$

where $K_{1}$ and $K_{2}$ are the curing reaction rate constants, m and n are the reaction order constants, D is the diffusion factor, and $\alpha_{c}$ is the critical degree of cure at the onset of diffusion effects.

The rate constants follow Arrhenius relations:

(16) $$K_{i}\left(T\right)=A_{i}exp\left(-\frac{\Delta E_{i}}{RT}\right),i=1,2,$$

where $A_{i}$ is the pre-exponential factor, $\Delta E_{i}$ is the activation energy, R is the universal gas constant, and T is the absolute temperature. The temperature dependence of $\alpha_{c}$ is represented by

(17) $$\alpha_{c}=\alpha_{c0}+\alpha_{cT}T.$$

The Kamal–Sourour parameters listed in Table 1 are taken from Ref. [20].

## 4. Numerical Implementation for Curing Simulation

A sequential thermo-chemo-mechanical finite element model is developed in Abaqus 2022 to predict the temperature and strain responses of E-51 epoxy resin during staged curing. The cure kinetics models provide the cure rate for calculating reaction heat generation in the thermal analysis and the degree of cure for evaluating cure-dependent mechanical properties and chemical shrinkage in the mechanical analysis.

### 4.1. Thermo-Chemo-Mechanical Formulation

The thermal–chemical calculation combines heat conduction with the reaction heat generated during cure. The heat-transfer equation and reaction heat source are written as:

(18) $$\rho C_{p}\frac{\partial T}{\partial t}=\nabla \cdot (k\nabla T)+q.$$

The volumetric heat-generation rate for resin curing can be expressed as follows:

(19) $$q=\rho \Delta H\frac{d\alpha }{dt},$$

where q is the volumetric heat-generation rate, ρ is the density of the resin, $C_{p}$ is the specific heat capacity, k is the thermal conductivity, and ΔH is the total reaction enthalpy.

Assuming that the specific heat capacity and thermal conductivity are linear functions of the degree of cure, they can be expressed by the following equations [21,22]:

(20) $$C_{p}=C_{p0}\left(1-\alpha \right)+C_{p1}\alpha ,$$

(21) $$k=k_{0}\left(1-\alpha \right)+k_{1}\alpha ,$$

The uncured and fully cured heat capacities and thermal conductivities in Table 2 are taken from the E-51 characterization reported in Ref. [20] and define the linear property relations in Equations (20) and (21). A constant resin density of 1108.9 kg/m^3^ is used in the thermal–chemical analysis.

The glass-transition temperature is related to the degree of cure using the E-51 parameters reported in Ref. [20], with a fitting parameter of *l* = 0.49 in the mechanical analysis:

(22) $$\frac{T_{g}-T_{g0}}{T_{g1}-T_{g0}}=\frac{l\alpha }{1-\left(1-l\right)\alpha },$$

where $T_{g}$ is the glass transition temperature at a degree of cure α, $T_{g0}$, and $T_{g1}$ are the glass transition temperatures of the uncured and fully cured resin, respectively, and l is the fitting parameter.

The evolution of the shear modulus during the curing process is described by the following equation [23], assuming the Poisson’s ratio of the resin is ν = 0.37:

(23) $$G=\left\{\begin{array}{ll} 0 & \alpha <\alpha_{gel}\\ G_{1} & T_{C}<T_{1}\\ G_{1}+\frac{T_{C}-T_{1}}{T_{2}-T_{1}}\left(G_{2}-G_{1}\right) & T_{1}\leq T_{C}<T_{2}\\ G_{2}+\frac{T_{C}-T_{2}}{T_{3}-T_{2}}\left(G_{3}-G_{2}\right) & T_{2}\leq T_{C}<T_{3}\\ G_{3}+\frac{T_{C}-T_{3}}{T_{4}-T_{3}}\left(G_{4}-G_{3}\right) & T_{3}\leq T_{C}<T_{4}\\ G_{4} & T_{C}\geq T_{4} \end{array}\right.,$$

The gel conversion, shear-modulus levels, and temperature-difference thresholds are taken from the E-51 characterization and constitutive approximation in Ref. [20]. The temperature difference is defined as

(24) $$T_{C}=T_{g}-T.$$

After gelation, the chemical shrinkage strain increment is proportional to the degree-of-cure increment:

(25) $$\Delta \varepsilon_{che}=\left\{\begin{array}{ll} 0, & 0\leq \alpha <\alpha_{gel}\\ {-E}_{che}\Delta \alpha , & \alpha_{gel}\leq \alpha \leq 1 \end{array}\right.,$$

where $E_{che}$ is the chemical shrinkage coefficient.

The thermal strain increment, $\Delta \varepsilon_{the}$, can be expressed as follows:

(26) $$\Delta \varepsilon_{the}=\left\{\begin{array}{ll} 0, & 0\leq \alpha <\alpha_{gel}\\ E_{the}\Delta T, & \alpha_{gel}\leq \alpha \leq 1 \end{array}\right.,$$

where $E_{the}$ is the coefficient of linear thermal expansion. The parameter values are listed in Table 3.

The same heat capacity, thermal conductivity, glass-transition relation, modulus, thermal expansion coefficient, and chemical shrinkage coefficient are used with both kinetic models.

### 4.2. Finite Element Model and Numerical Implementation

**Figure 3:**
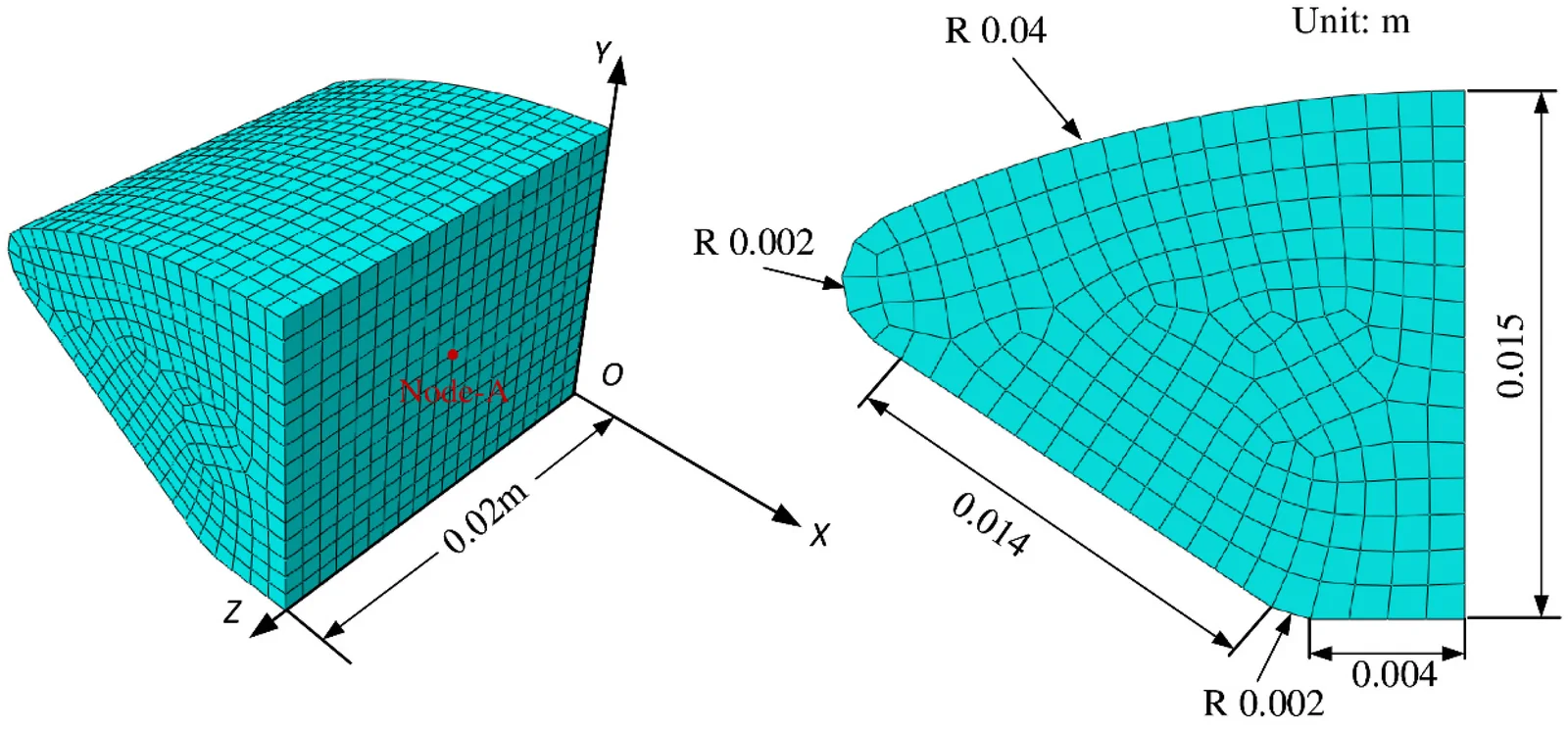
Geometry and finite element mesh of the E-51 epoxy resin encapsulation structure.

A three-dimensional finite element model of the E-51 encapsulation structure is constructed using the dimensions in Figure 3. Symmetry about the YOZ plane allows half of the structure to be modeled. Eight-node DC3D8 and C3D8 elements are used for the thermal–chemical and mechanical analyses, respectively. Both analyses extend to 102,600 s, with a maximum time increment of 100 s.

A sequential coupling scheme is adopted, assuming that deformation has a negligible influence on the thermal–chemical response. UMATHT evaluates the local cure rate, updates the degree of cure, and calculates reaction heat from the accepted conversion increment. The nodal temperature history is transferred to the mechanical analysis as a predefined field. UEXPAN integrates the local kinetics along this history to determine conversion, thermal expansion, and chemical shrinkage, while UMAT evaluates stiffness and stress. Table 4 summarizes the functions of the user subroutines.

In the thermal–chemical analysis, the symmetry plane is adiabatic, and the temperature history specified in Section 2.3 is prescribed on the remaining external surfaces through DISP. The same thermal boundary conditions are used for both kinetics models. During cooling, the prescribed surface temperature follows an expression fitted to the cooling measurements:

(27) $$T=T_{r}+\left(T_{s}-T_{r}\right)C^{\left(t-t_{s}\right)},$$

where T is the temperature at time t, $T_{s}$ and $T_{r}$ are the initial and asymptotic final temperatures of the cooling stage, respectively, $t_{s}$ is the cooling onset time, and C = 0.9995 is a fitting parameter, with t and $t_{s}$ expressed in seconds. Here, $T_{s}$ = 70 °C, $T_{r}$ = 25 °C, and $t_{s}$ = 98,400 s.

In the mechanical analysis, X symmetry is imposed on the YOZ plane, displacement normal to the XOY plane is constrained on the bottom surface, and point O is fixed. The temperature, X-direction strain, and degree-of-cure histories are extracted at monitoring point A.

During numerical integration, the cure rate is constrained to be nonnegative, and the conversion increment is limited to the remaining uncured fraction to maintain the degree of cure within [0, 1]. For Dual Kriging, local temperatures above 70 °C are evaluated by extrapolating the rate relation beyond the measured DSC temperature range.

## 5. Results and Discussion

### 5.1. DSC Characterization and Modeling Data

At 10, 15, and 20 °C/min, the peak temperatures are 115.711, 124.737, and 133.791 °C, respectively, and the corresponding integrated reaction heats are 392.3, 410.1, and 404.7 J/g [20]. The exothermic peak shifts toward higher temperatures as the heating rate increases.

**Figure 4:**
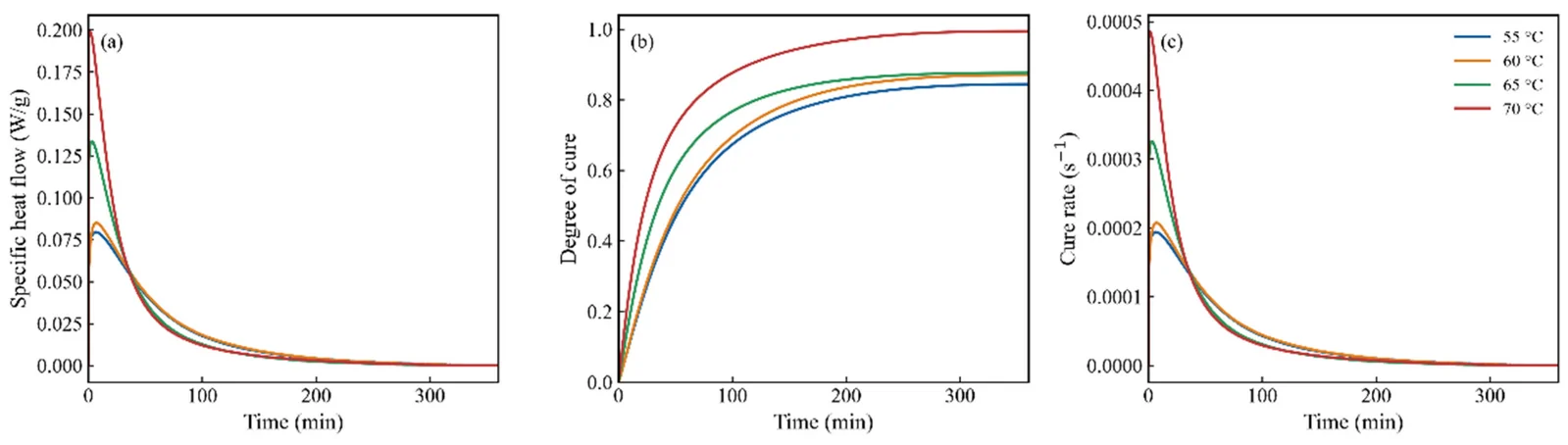
Isothermal DSC data: (**a**) specific heat flow; (**b**) degree of cure; (**c**) cure rate. Conversion and rate are calculated using a reference reaction heat of 410.1 J/g.

Figure 4 presents the isothermal DSC heat-flow curves and the corresponding degree-of-cure and cure-rate histories. The isothermal reaction heats at 55, 60, 65, and 70 °C are 346.4, 357.1, 359.7, and 408.0 J/g, respectively. Using the common reference reaction heat of 410.1 J/g defined in Section 3.1, the corresponding degrees of cure after 360 min are 0.84457, 0.87070, 0.87714, and 0.99479. Within the investigated temperature range, higher curing temperatures result in greater recorded reaction heat and higher conversion at the end of the holding period, indicating the influence of temperature on the reaction progress over the prescribed curing time.

### 5.2. Data Compression and Model Selection

#### 5.2.1. Accuracy of the Compressed Representation

Table 5 summarizes the compression of the combined isothermal dataset using linear drift and a linear covariance kernel. The initial set contains 12 points. Successive additions of points at local error maxima increase the retained point count to 16, 36, 81, and 187, while reducing the maximum reconstruction error from 61.93% to 0.45%. The final representation satisfies the prescribed 0.5% tolerance and reduces the dataset from 7200 to 187 points, corresponding to a reduction of 97.40%. The mean reconstruction error is 0.069%.

**Figure 5:**
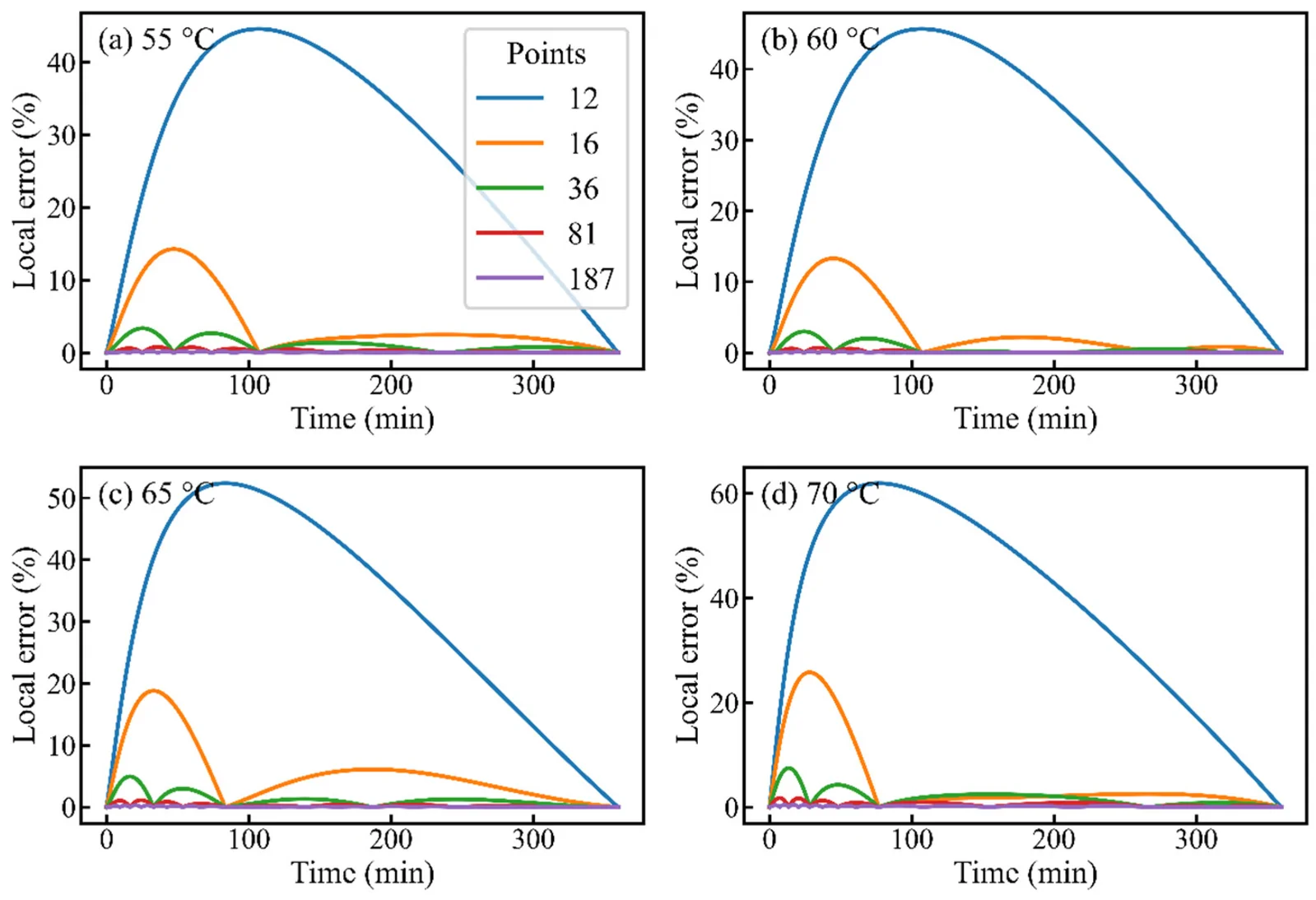
Local reconstruction errors during compression of the isothermal DSC data: (**a**) 55 °C; (**b**) 60 °C; (**c**) 65 °C; and (**d**) 70 °C. Legend entries give the total retained point count.

The local error distributions in Figure 5 show that the initial representation does not adequately resolve the curved portions of the isothermal histories. Adding points at local error maxima progressively reduces these deviations. The final mean error is substantially lower than the maximum error, indicating that the remaining deviations are localized rather than distributed uniformly along the curves. The results demonstrate that most of the densely sampled observations can be removed while retaining the measured degree-of-cure profiles within the prescribed tolerance.

#### 5.2.2. Drift–Covariance Selection and Computational Cost

The admissible drift–covariance combinations are compared using leave-one-out cross-validation on the same 187-point dataset. Table 6 lists the cumulative, mean, and maximum absolute errors. For both linear and quadratic drift, the cubic covariance kernel produces lower errors than the linear and logarithmic kernels. With linear drift, changing from the linear to the cubic kernel reduces the mean absolute error from 2.699 × 10^−3^ to 3.640 × 10^−4^, a reduction of approximately 86.5%.

Quadratic drift with cubic covariance gives an MAE approximately 1.9% lower than linear drift with cubic covariance, based on the rounded values in Table 6. Linear drift is selected because it provides comparable reconstruction accuracy with fewer polynomial terms.

**Figure 6:**
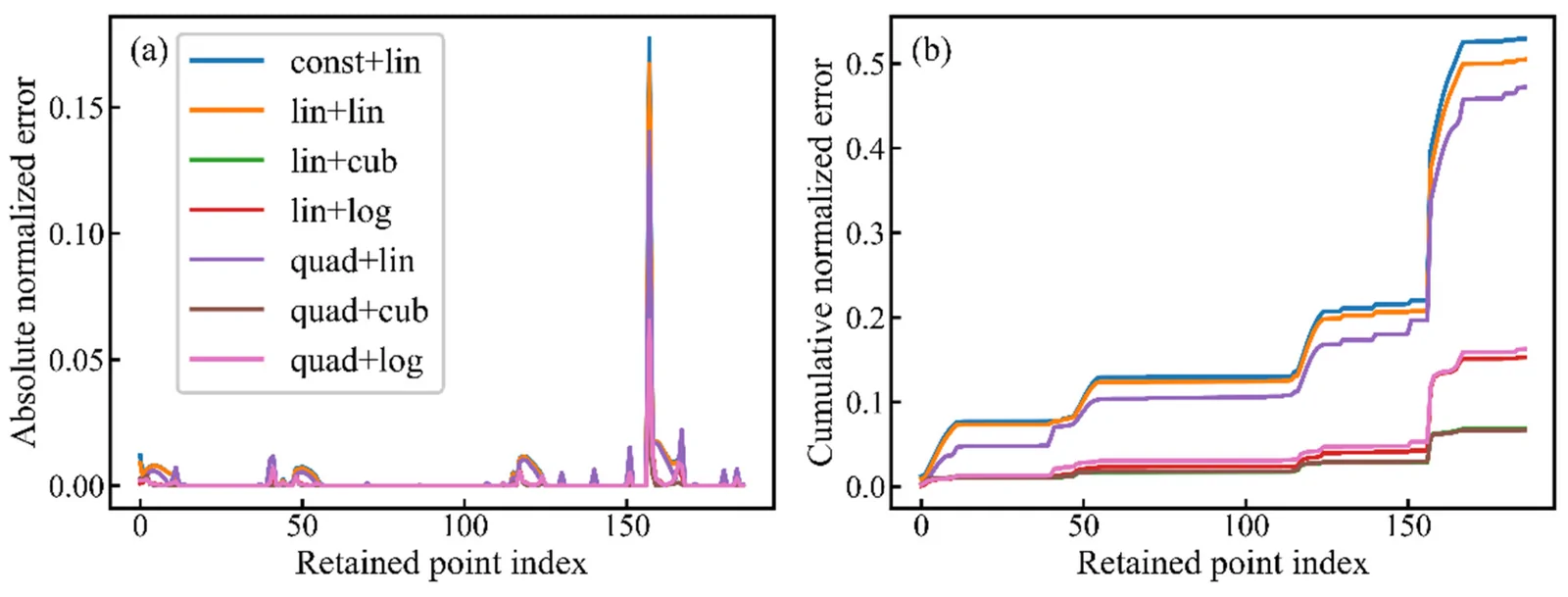
Leave-one-out cross-validation errors for the 187-point isothermal dataset: (**a**) local absolute error; and (**b**) cumulative absolute error.

Figure 6 shows the local and cumulative leave-one-out errors for the candidate configurations on the fixed 187-point dataset.

**Figure 7:**
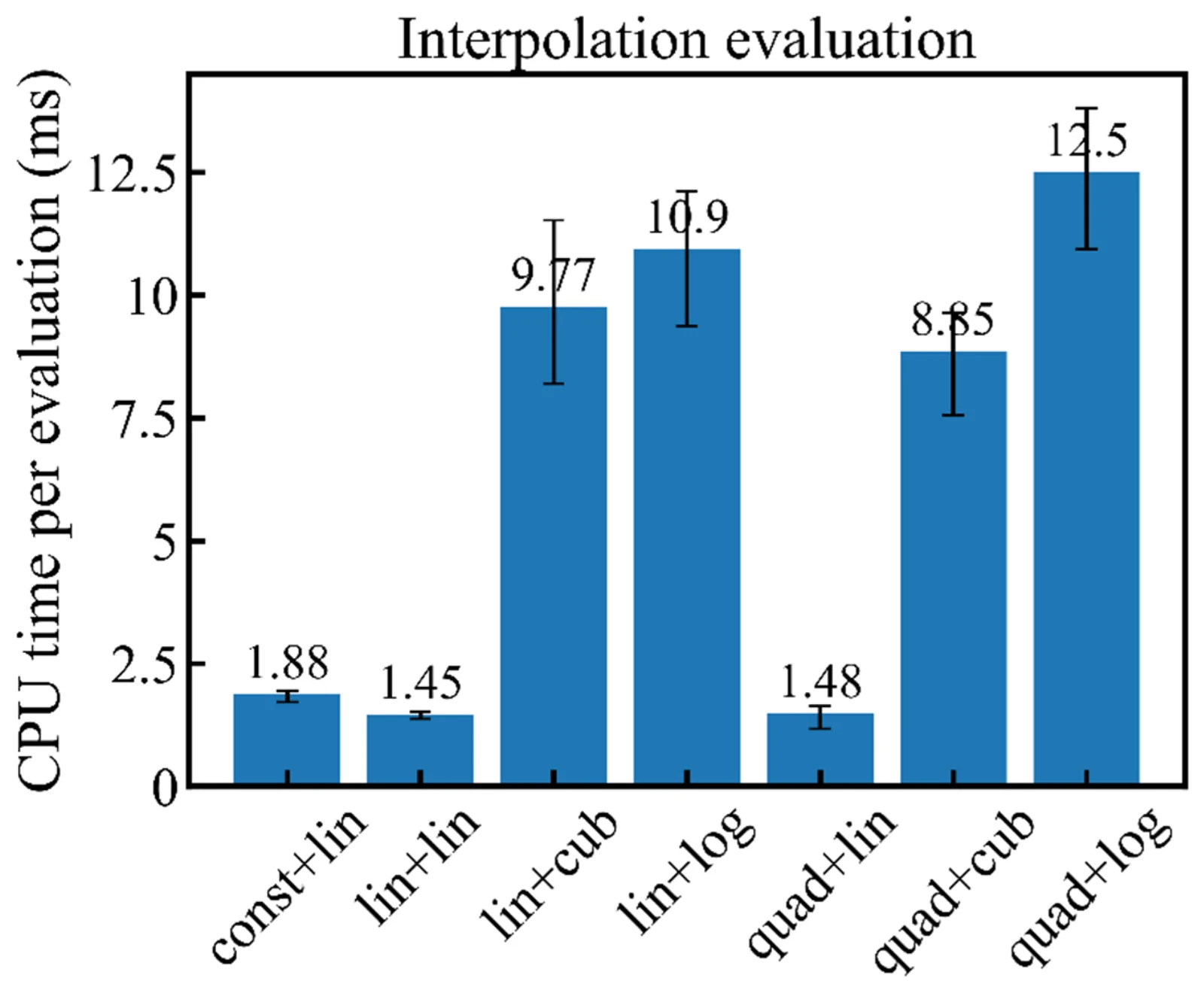
CPU time for evaluating the constructed degree-of-cure interpolants at the 7200 original isothermal data points. Bars show the median time per evaluation, and error bars indicate the interquartile range across repeated batches.

Figure 7 compares the CPU time required to evaluate the constructed interpolants at the 7200 original data points. The measurements were performed on a computer equipped with an Intel Core i9-14900KF processor. Evaluation times are approximately 1.45–1.88 ms for the linear kernel, 8.85–9.77 ms for the cubic kernel, and 10.9–12.5 ms for the logarithmic kernel. The cubic kernel therefore provides lower cross-validation errors at a higher evaluation cost than the linear kernel in this implementation. Its selection reflects the balance between reconstruction accuracy and computational cost.

Linear drift with cubic generalized covariance is therefore also used for the local cure-rate representation in Section 3.4.

For the 245-node local rate surface, reconstruction of the experimental rate data gives RMSE values of 7.217 × 10^−6^, 6.493 × 10^−6^, 9.113 × 10^−6^, and 1.101 × 10^−5^ s^−1^ at 55, 60, 65, and 70 °C, respectively. These values quantify reconstruction of the contributing rate data; prediction at excluded temperatures is evaluated in Section 5.5.

### 5.3. Comparison with the Kamal–Sourour Model

**Figure 8:**
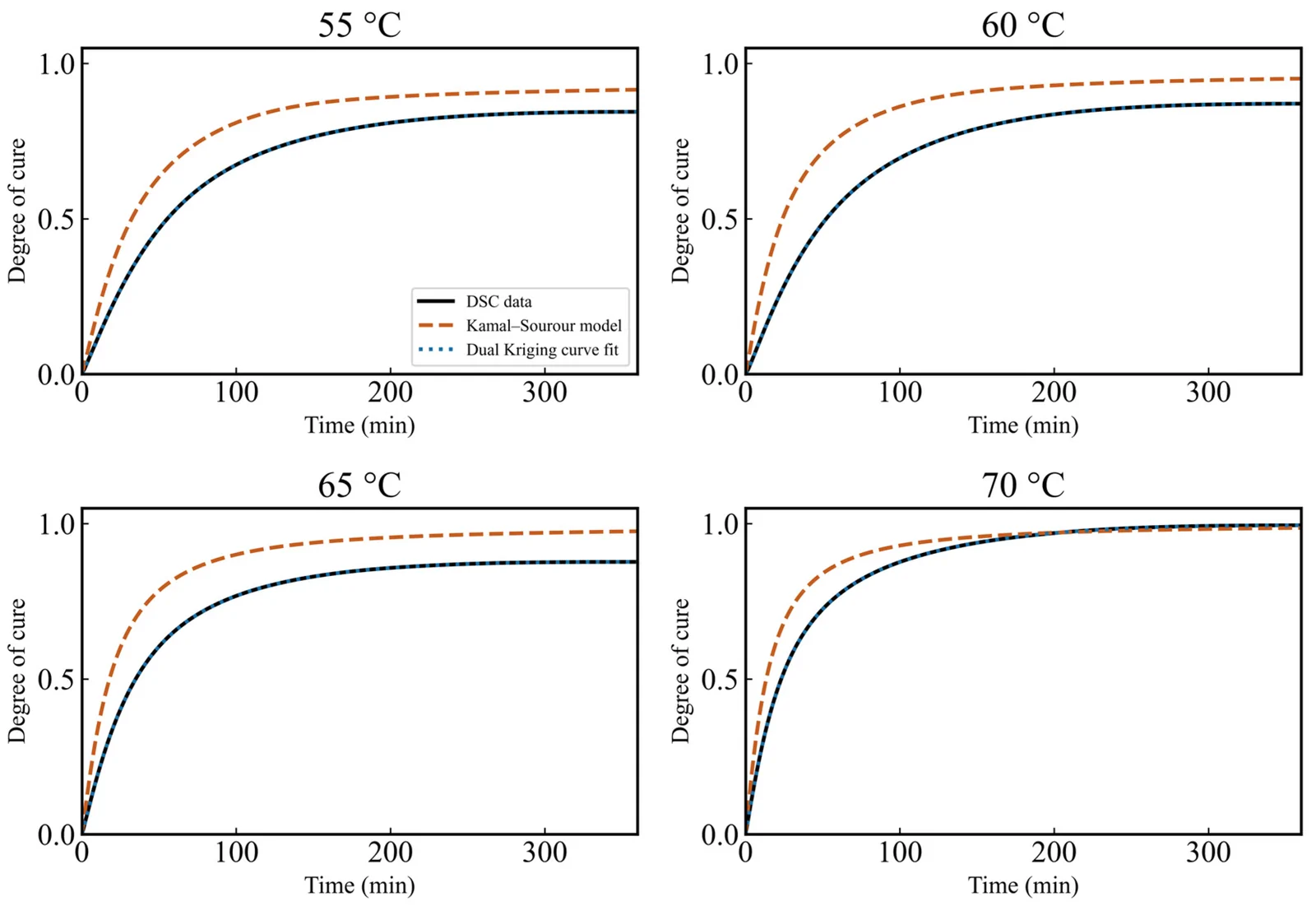
Isothermal DSC conversion histories, integrated Kamal–Sourour predictions, and Dual Kriging reconstructions at 55, 60, 65, and 70 °C.

Figure 8 compares the measured isothermal conversion histories with the Kamal–Sourour reference trajectories and the 187-point Dual Kriging reconstructions. The Kamal–Sourour trajectories are obtained by integrating Equations (15)–(17) with the fixed parameters in Table 1, whereas the Dual Kriging curves are reconstructed directly from the measured time–temperature data using the conversion interpolant.

At 55–65 °C, the Kamal–Sourour model overestimates the degree of cure during most of the holding period, with deviations persisting as the curves approach their final plateaus. At 70 °C, the model also overestimates the initial cure evolution, but the difference decreases with time, and the final degree of cure approaches the measured value. These results indicate that the temperature-dependent parameter set in Table 1 does not reproduce all four isothermal histories with the same accuracy.

The Dual Kriging curves closely follow the DSC data at all four temperatures, capturing both the initial increase in the degree of cure and the subsequent plateau. This agreement demonstrates the accuracy of the compressed interpolation representation for the measured isothermal curves.

### 5.4. Comparison with FBG Measurements and Interpretation of Cure Evolution

**Figure 9:**
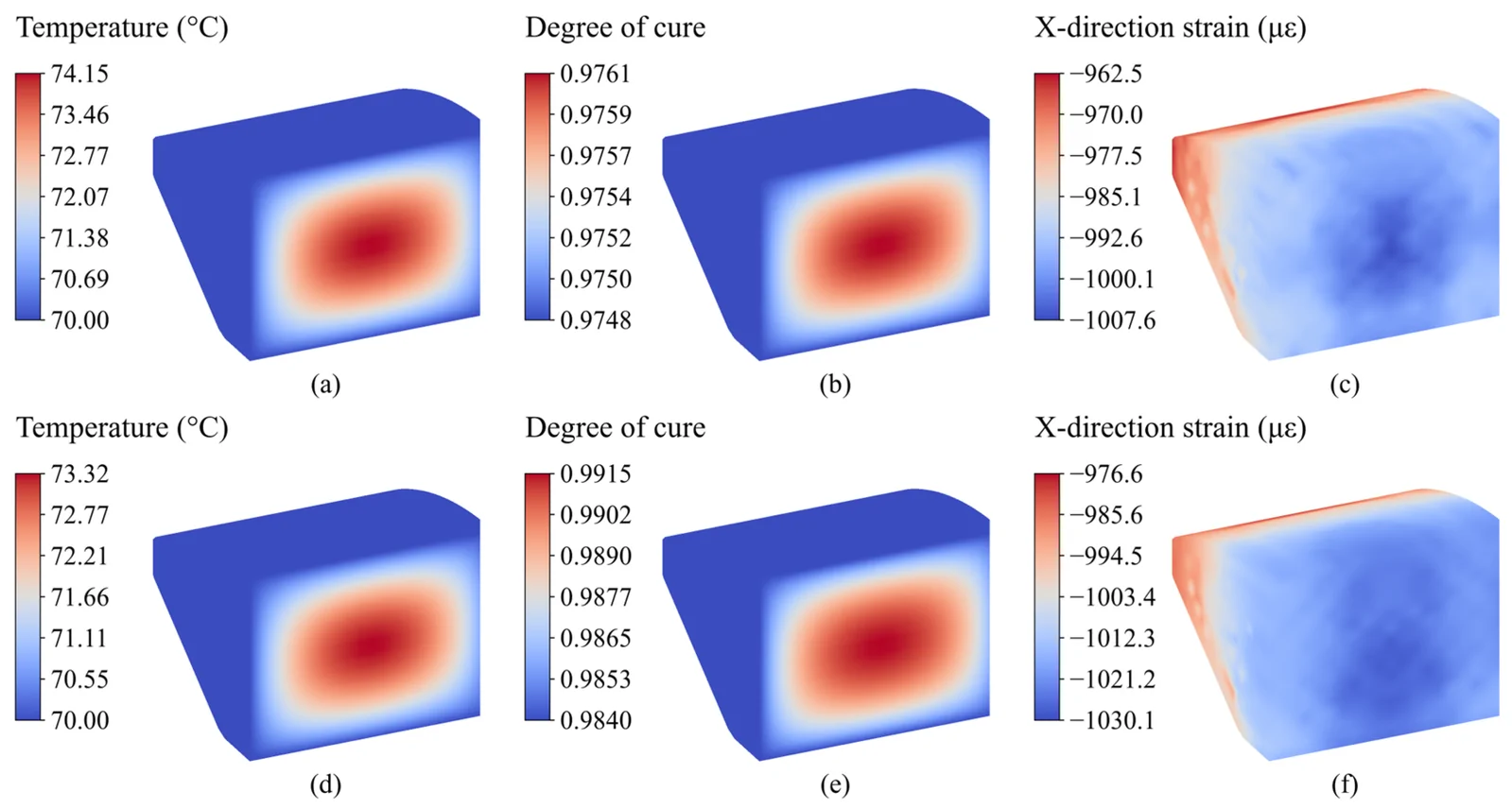
FE fields for Kamal–Sourour (**a**–**c**) and Dual Kriging (**d**–**f**): temperature at each model’s point-A peak; final degree of cure; final X-direction strain. Conversion and strain are shown as element-nodal averages. Each panel uses its own color scale.

Figure 9 presents the temperature, degree-of-cure, and X-direction strain distributions calculated using the Kamal–Sourour and Dual Kriging models. Both models predict nonuniform temperature fields, with higher temperatures in the interior of the resin structure. The degree-of-cure distributions exhibit corresponding spatial variations, while the strain fields reflect the combined effects of thermal deformation, chemical shrinkage, and mechanical constraints.

**Figure 10:**
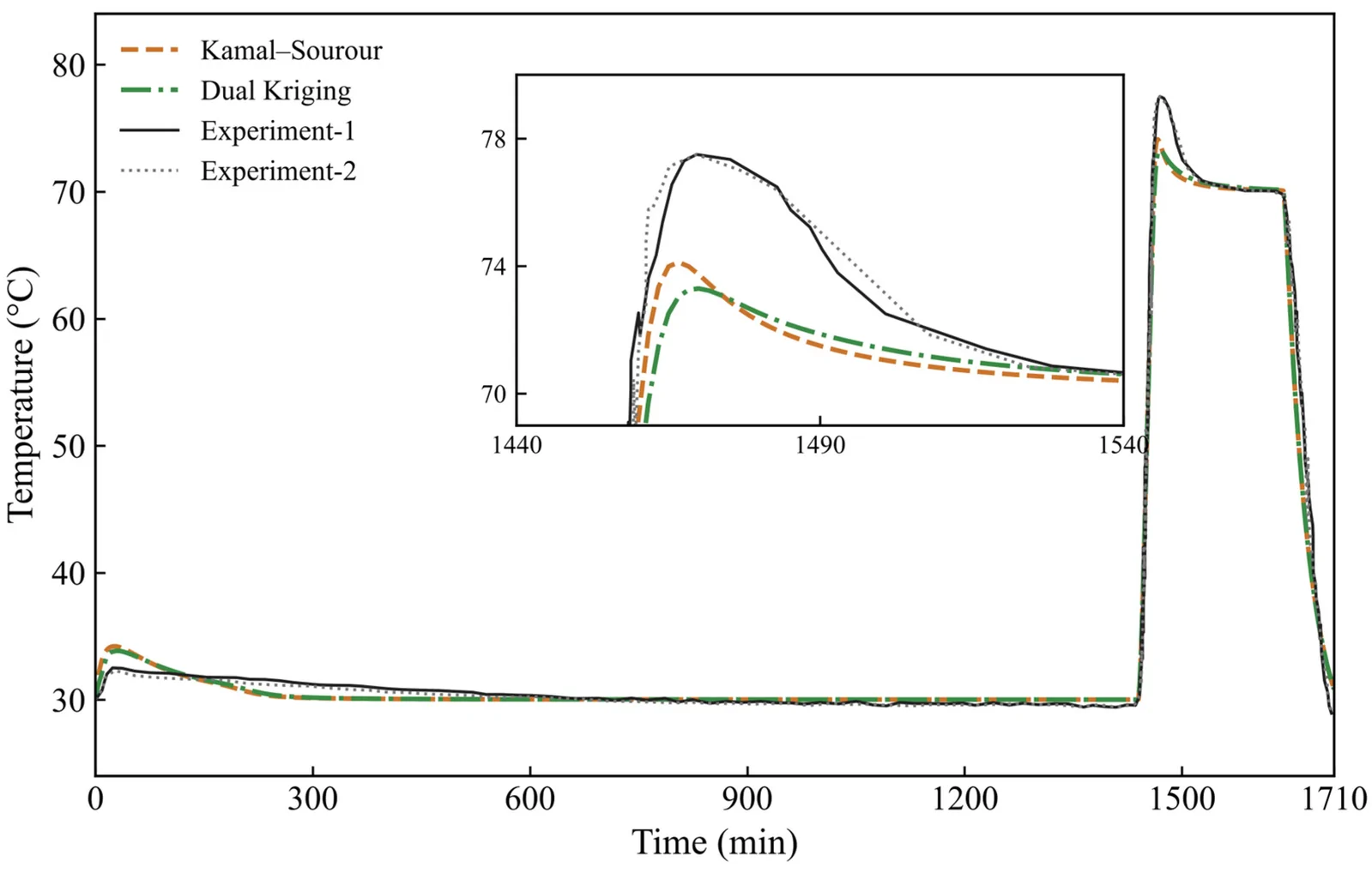
Predicted and FBG-measured temperatures at point A; inset: peak-temperature region.

Figure 10 compares the predicted and FBG-measured temperature histories at point A. The predicted temperature peaks at point A are 74.12 °C for Kamal–Sourour and 73.29 °C for Dual Kriging, compared with 77.50 °C in both experiments. The corresponding absolute peak-temperature errors are 3.38 and 4.21 °C, respectively.

**Figure 11:**
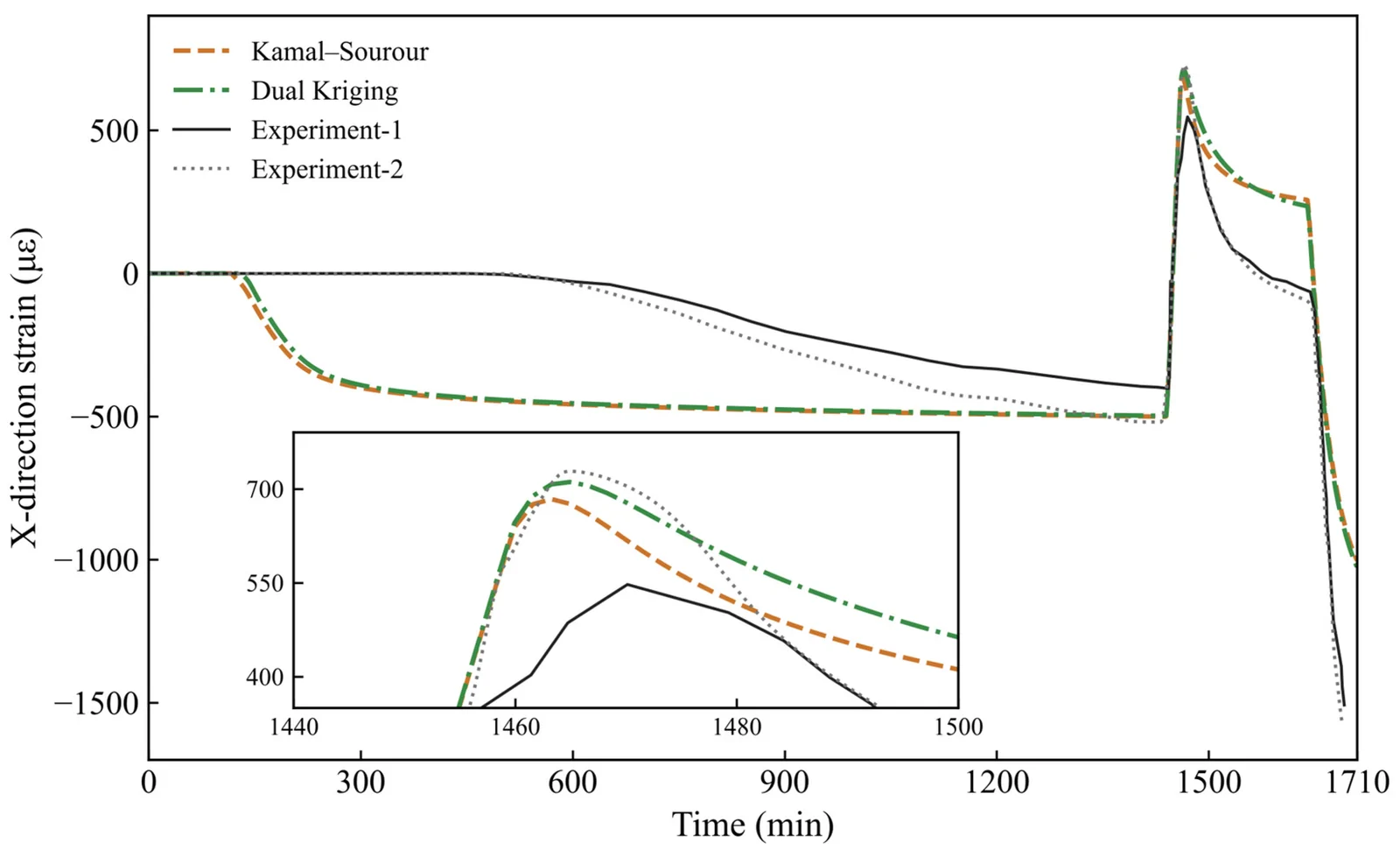
Predicted and FBG-measured X-direction strains at point A; inset: peak-strain region. Experiment-2 is smoothed for display.

Figure 11 compares the predicted and FBG-measured X-direction strain histories at point A. At the simulation end time of 1710 min, the predicted strains at point A are −1003.84 and −1026.13 με for Kamal–Sourour and Dual Kriging, respectively. The experimental records end at 1691.935 and 1689.033 min, with measured strains of −1508.21 and −1574.12 με. At these respective times, Kamal–Sourour predicts −872.31 and −843.68 με, while Dual Kriging predicts −894.61 and −865.99 με. Both models therefore underestimate the measured contraction at the corresponding observation times.

A one-factor sensitivity analysis is performed using the same Dual Kriging temperature history and mechanical constraints. Increasing the chemical shrinkage coefficient and thermal expansion coefficient separately by 10% changes the final strain at point A by −101.08 and −1.53 με, respectively. Raising both glass-transition endpoints by 5 K changes the final strain by −15.64 με, while increasing all shear-modulus levels by 10% produces no change at the reported precision. Among these prescribed perturbations, the chemical shrinkage coefficient has the largest influence on the final strain.

**Figure 12:**
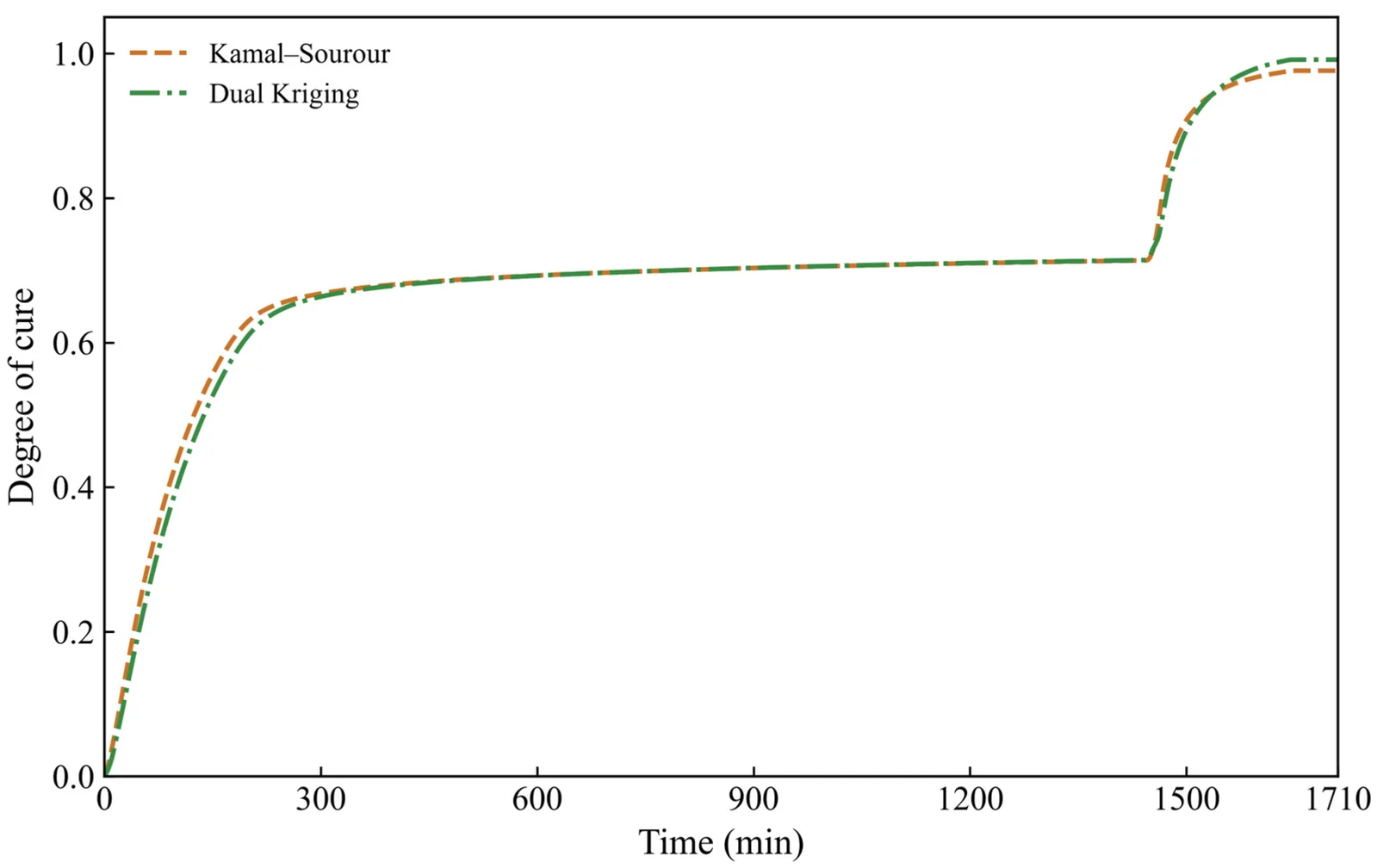
Simulated degree-of-cure histories at point A.

Figure 12 presents the simulated conversion histories at point A. The final degrees of cure are 0.97608 for Kamal–Sourour and 0.99141 for Dual Kriging. At the end of the initial 24 h dwell at 30 °C, the corresponding degrees of cure are 0.71362 and 0.71408. Both models use the same Kamal–Sourour-generated low-temperature kinetics during this stage and enter the subsequent heating stage with similar conversion and remaining reaction heat.

The relative contributions of the thermal boundary, material properties, low-temperature kinetic inputs, and FBG strain transfer to the remaining temperature and strain discrepancies have not been separately quantified.

### 5.5. Validation Using Independent DSC Data

The Dual Kriging rate model is evaluated using a public DSC dataset for a DGEBA/amine epoxy system [24] and its associated study [25]. Seven isothermal measurements from 40 to 100 °C, at intervals of 10 °C, provide the experimental basis for constructing the cure-rate surface. The apparent degree of cure is calculated from the recorded reaction heat using a reference enthalpy of 470 J/g, without compensation for unrecorded initial heat.

The entire 70 °C curve is withheld from model construction. Three training sets are considered: 40/100 °C, 40/60/80/100 °C, and 40/50/60/80/90/100 °C. Each training curve is represented by 35 uniformly spaced apparent-conversion nodes, using linear drift and cubic generalized covariance. All three models are evaluated against the same 1123 observations over an apparent-conversion interval of 0.05–0.806216 and a time interval of 5.1–117.3 min. Model settings are fixed independently of the test errors. Rate RMSE is normalized by the maximum measured rate within this interval.

**Figure 13:**
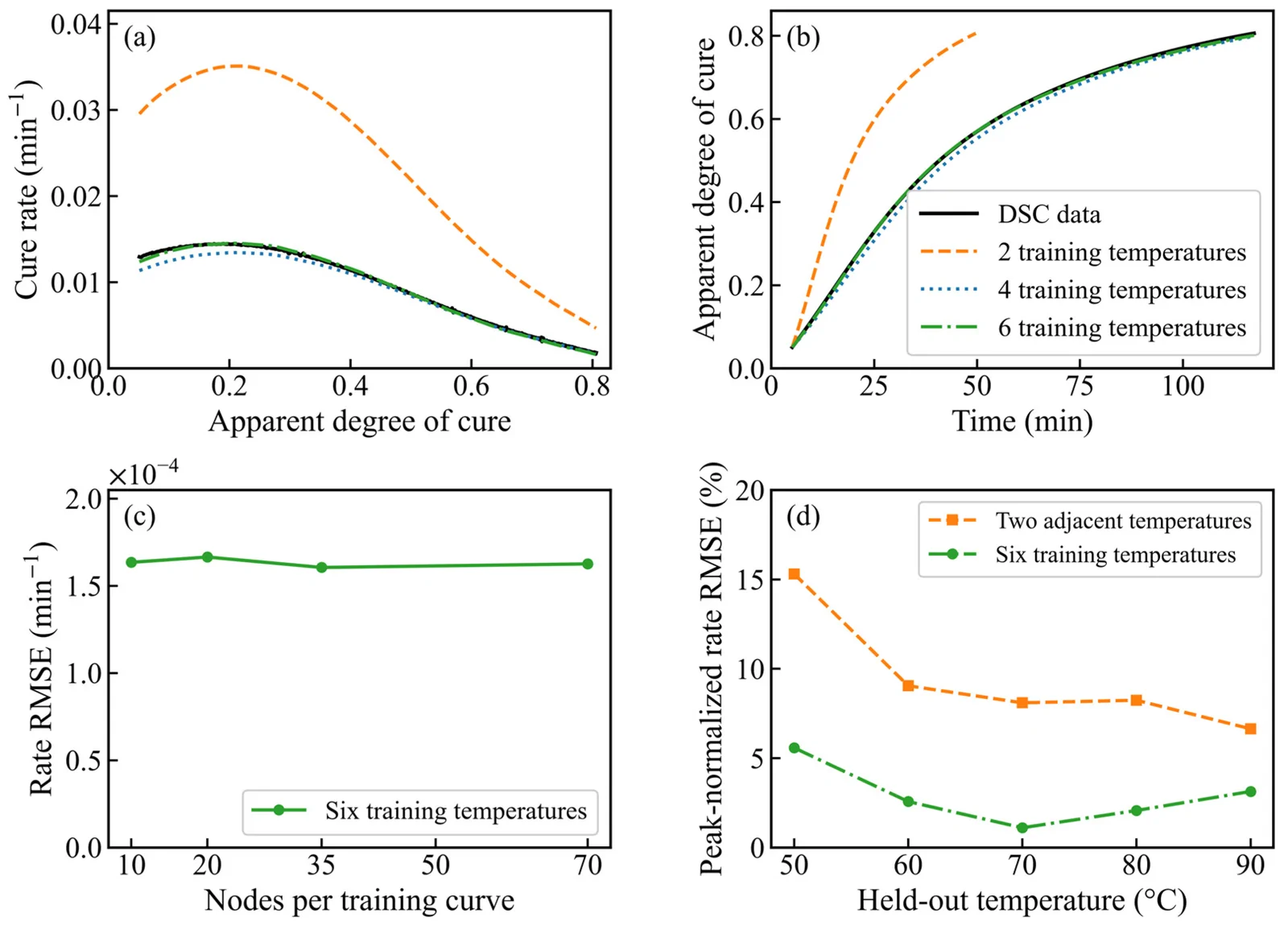
Validation using independent DSC data [24]: (**a**) cure-rate prediction at the held-out temperature of 70 °C; (**b**) corresponding apparent-conversion histories; (**c**) effect of nodes per curve with six training temperatures; (**d**) rate prediction errors at five held-out temperatures.

Table 7 and Figure 13a show progressively closer agreement with the measured rate profile as the training temperature set is expanded. The peak-normalized rate RMSE decreases from 81.12% for two training temperatures to 3.44% for four and 1.11% for six. These results assess prediction at a temperature excluded from model construction.

Integration of the predicted rates gives the apparent-conversion histories in Figure 13b, initialized from the first measured value. The four- and six-temperature models cover the full evaluation interval, with apparent-conversion RMSE values of 0.01422 and 0.00271, respectively. The two-temperature trajectory reaches the upper boundary of the training domain at 49.763 min, covering only 39.81% of the evaluation interval. Its apparent-conversion RMSE of 0.23095 therefore applies to a shorter interval and is not directly comparable with the full-interval values.

To assess interpolation across the temperature range, each of the five interior curves is withheld in turn, and the model is constructed from either the two adjacent temperatures or all six remaining temperatures. The six-temperature models give lower rate errors in all five cases, with peak-normalized RMSE values of 1.11–5.58% (Table 8 and Figure 13d). At 70 °C, the adjacent 60/80 °C pair gives an error of 8.10%, compared with 81.12% for the more widely separated 40/100 °C pair. Thus, the distribution of the training temperatures, as well as their number, affects interpolation accuracy.

The influence of sampling density along each curve is examined while retaining the six training temperatures. Increasing the number of nodes per curve from 10 to 70 yields a 70 °C rate RMSE of 1.606 × 10^−4^–1.666 × 10^−4^ min^−1^, with no systematic reduction (Figure 13c). Denser sampling of the existing curves therefore provides little additional improvement for this configuration.

For comparison with an established kinetic equation, the diffusion-kinetic model and recommended parameters reported in the associated study [25] are adopted:

(28) $$\frac{d\alpha }{dt}=\frac{A\;exp(-E_{a}/RT)\alpha^{m}{\left(1-\alpha \right)}^{n}}{1+exp\{C[\alpha -\alpha_{c}(T)]\}},\;\alpha_{c}(T)=aT+b$$

The published parameters are A = 2.50 × 10^5^ s^−1^, $E_{a}$ = 56.24 kJ mol^−1^, m = 0.41, n = 1.83, C = 43.7, a = 5.27 × 10^−3^ K^−1^, and b = −0.885, with T in kelvin and t in seconds. Rates are converted to min^−1^ for comparison.

**Figure 14:**
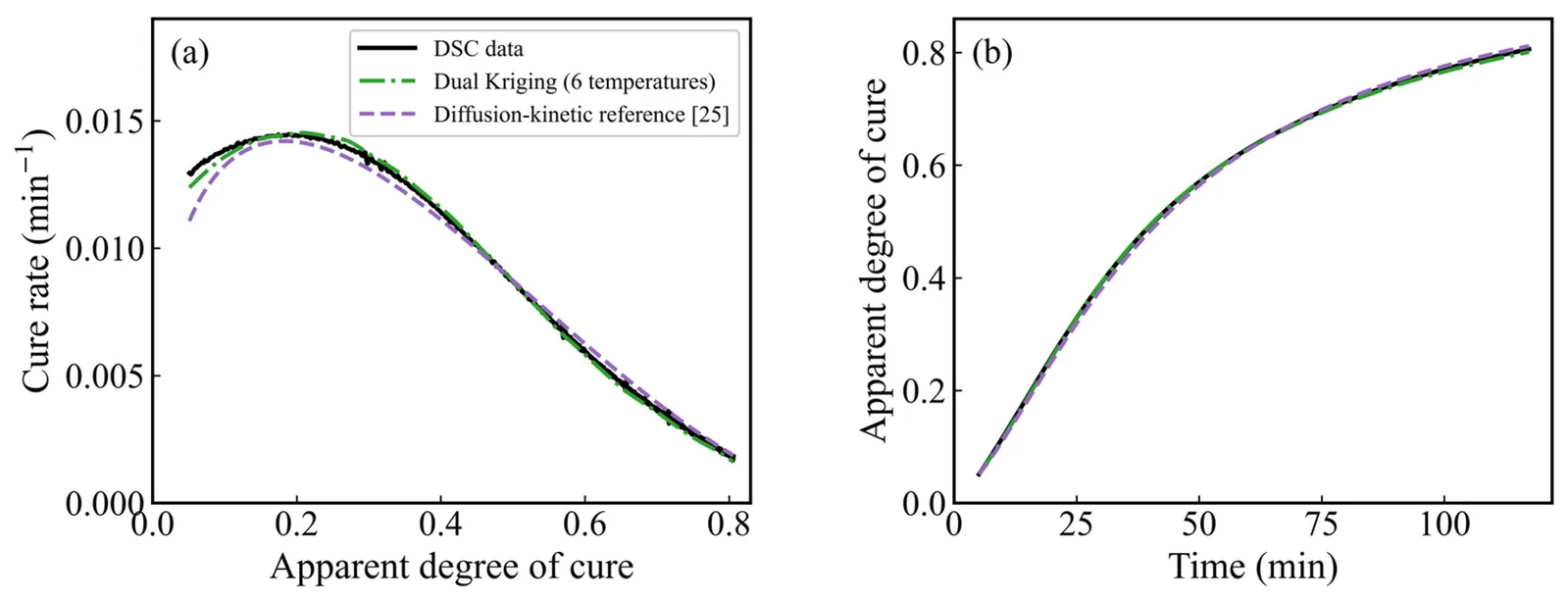
Comparison with the diffusion-kinetic reference [25] at 70 °C: (**a**) cure rate; (**b**) apparent-conversion history.

Figure 14 compares the literature model with the six-temperature Dual Kriging model at 70 °C. Both are evaluated over the same interval, and their apparent-conversion histories start from the same measured initial value. The literature model gives a peak-normalized rate RMSE of 2.30% and an apparent-conversion RMSE of 0.00617, compared with 1.11% and 0.00271 for Dual Kriging. The published parameters were fitted using data that included 70 °C and are applied here without refitting, whereas the entire 70 °C curve is withheld from Dual Kriging training.

The complete-curve holdouts support the temperature-interpolation capability of the Dual Kriging rate model for this resin within the evaluated apparent-conversion range. With six training temperatures, the model reproduces both the rate profile and the integrated apparent-conversion history at 70 °C, with lower errors than the adopted literature reference under the stated comparison conditions.

## 6. Conclusions

A Dual Kriging framework combining error-controlled DSC-data compression and a separable local cure-rate representation was developed and implemented in sequential thermo-chemo-mechanical FE analysis. The main conclusions are as follows:

1. The E-51 isothermal conversion dataset was reduced from 7200 to 187 points, a reduction of 97.40%, with maximum and mean normalized reconstruction errors of 0.45% and 0.069%. The reconstructed curves retained the measured initial cure evolution and late-stage plateaus. With linear drift, the cubic covariance kernel reduced the mean leave-one-out error by 86.5% relative to the linear kernel.
2. Independent DSC validation demonstrated prediction at temperatures excluded from model construction. With six experimental training temperatures, the withheld 70 °C curve gave a peak-normalized rate RMSE of 1.11% and an apparent-conversion RMSE of 0.00271. The corresponding errors for the adopted literature model were 2.30% and 0.00617, respectively; its published parameters had been fitted using data that included 70 °C.
3. Training-temperature coverage had a stronger effect than curve-node density in the independent dataset. Expanding the training set from four to six temperatures reduced the 70 °C rate error from 3.44% to 1.11%, whereas increasing the nodes per curve from 10 to 70 at six fixed training temperatures produced little change. Six-temperature models gave peak-normalized rate RMSE values of 1.11–5.58% across the five withheld interior temperatures.
4. The 245-node local rate representation was implemented in the E-51 FE analysis using experimental and auxiliary Kamal–Sourour inputs. Dual Kriging and Kamal–Sourour produced similar temperature and strain histories, with predicted temperature peaks of 73.29 and 74.12 °C, respectively, compared with 77.50 °C experimentally. Both models underestimated the measured contraction at the corresponding observation times. The final simulated degrees of cure were 0.99141 and 0.97608, respectively.

The present E-51 application is limited by the number and temperature coverage of the available DSC measurements, particularly below 55 °C. Future work will expand the experimental dataset across the temperature range encountered during curing, including the initial low-temperature dwell and temperatures reached through reaction self-heating. These measurements will provide direct inputs for the local rate model and support improved predictions under practical curing schedules. Validation under additional non-isothermal curing schedules will further assess the model’s predictive capability under different thermal histories.

## Figures and Tables

**Table 1:** Kamal–Sourour model parameters.

Parameter	Value
$A_{1}$/s^−1^	9.64 × 10^6^
$A_{2}$/s^−1^	1.36 × 10^3^
$\Delta E_{1}$/(J⋅mol^−1^)	6.69 × 10^4^
$\Delta E_{2}$/(J⋅mol^−1^)	4.12 × 10^4^
m	0.3
n	1.7
D	50
$\alpha_{c0}$	−2.40
$\alpha_{cT}$/K^−1^	0.01

**Table 2:** Thermophysical parameters used in the FE model; property values from Ref. [20] and the common reaction-heat reference defined in Section 3.1.

Parameter	Value
ΔH/(J⋅g^−1^)	410.1
ρ/(kg⋅m^−3^)	1108.9
$k_{0}$/(W/(m⋅K))	0.16
$k_{1}$/(W/(m⋅K))	0.22
$C_{p0}$/(J/(kg⋅K))	1763
$C_{p1}$/(J/(kg⋅K))	1171

**Table 3:** E-51 constitutive parameters used in the mechanical implementation, consistent with Ref. [20].

Parameter	Value
$T_{g0}$/°C	−47.78
$T_{g1}$/°C	84.03
l	0.49
$\alpha_{gel}$	0.48
$T_{1}$/K	−5
$T_{2}$/K	5
$T_{3}$/K	15
$T_{4}$/K	50
$G_{1}$/Pa	1.35 × 10^6^
$G_{2}$/Pa	4.25 × 10^8^
$G_{3}$/Pa	7.65 × 10^8^
$G_{4}$/Pa	1.045 × 10^9^
$E_{che}$	2.0 × 10^−3^
$E_{the}$/K^−1^	32.0 × 10^−6^

**Table 4:** User subroutines and their functions in the curing analysis.

Subroutine	Function
UMATHT	Define the heat release during resin curing
DISP	Define the temperature boundary conditions
UEXPAN	Reintegrate cure and define thermal expansion and chemical shrinkage
UMAT	Define the stiffness matrix and stress-strain constitutive relationship
SDVINI	Define the initial values of state variables

**Table 5:** Reconstruction errors during compression of the isothermal dataset using linear drift and a linear covariance kernel.

Iteration	Original Points	Retained Points	Maximum Error (%)	Mean Error (%)	Data Reduction (%)
0	7200	12	61.93	31.83	99.83
1	7200	16	25.76	4.42	99.78
2	7200	36	7.47	1.16	99.50
3	7200	81	1.75	0.29	98.88
4	7200	187	0.45	0.069	97.40

Note: Errors in the normalized degree of cure are reported as percentages.

**Table 6:** Leave-one-out cross-validation errors for the 187-point isothermal dataset.

Drift	Covariance Kernel	Cumulative Absolute Error	Mean Absolute Error	Maximum Absolute Error
Constant	Linear	0.53	2.832 × 10^−3^	0.18
Linear	Linear	0.50	2.699 × 10^−3^	0.17
Linear	Cubic	0.068	3.640 × 10^−4^	0.029
Linear	Logarithmic	0.15	8.160 × 10^−4^	0.073
Quadratic	Linear	0.47	2.525 × 10^−3^	0.14
Quadratic	Cubic	0.067	3.570 × 10^−4^	0.028
Quadratic	Logarithmic	0.16	8.680 × 10^−4^	0.065

Note: All errors are dimensionless and are calculated from the normalized degree of cure.

**Table 7:** Prediction errors for the excluded 70 °C curve using 35 nodes per training curve. Rate NRMSE is normalized by the measured peak rate. Apparent-conversion RMSE is evaluated over the time interval covered by each integrated prediction.

Training Temperatures	Rate RMSE (min^−1^)	Rate NRMSE (% of Peak)	Apparent Degree of Cure RMSE	Time-Window Coverage (%)
2	0.0117459	81.12	0.23095	39.81
4	0.0004987	3.44	0.01422	100.00
6	0.0001606	1.11	0.00271	100.00

**Table 8:** Peak-normalized rate RMSE at the five interior temperatures. Each test curve is excluded from the corresponding training set.

Held-Out Temperature (°C)	Test Observations	Two Adjacent Temperatures (%)	Six Training Temperatures (%)
50	3635	15.31	5.58
60	1854	9.05	2.58
70	1123	8.10	1.11
80	651	8.24	2.08
90	359	6.64	3.14

## Data Availability

The E-51 data are available on request from the corresponding author. The public DSC dataset used for independent validation is available at https://doi.org/10.5281/zenodo.8113838 [24].

## References

[1] Wisnom M.R., Gigliotti M., Ersoy N., Campbell M., Potter K.D. (2006). Mechanisms generating residual stresses and distortion during manufacture of polymer-matrix composite structures. Compos Part A Appl. Sci. Manuf..

[2] Bogetti T.A., Gillespie J.W. (1992). Process-induced stress and deformation in thick-section thermoset composite laminates. J. Compos Mater..

[3] Johnston A., Vaziri R., Poursartip A. (2001). A plane strain model for process-induced deformation of laminated composite structures. J. Compos Mater..

[4] Svanberg J.M., Holmberg J.A. (2004). Prediction of shape distortions. Part I. FE-implementation of a path dependent constitutive model. Compos Part A Appl. Sci. Manuf..

[5] Svanberg J.M., Holmberg J.A. (2004). Prediction of shape distortions. Part II. Experimental validation and analysis of boundary conditions. Compos Part A Appl. Sci. Manuf..

[6] Sourour S., Kamal M.R. (1976). Differential scanning calorimetry of epoxy cure: Isothermal cure kinetics. Thermochim. Acta.

[7] Corsetti R.R., Neumeyer T., May M., Jandrey D., Altstaedt V., Cardozo N.S.M. (2013). Modeling and estimation of parameters for the curing of an epoxy/amine system. Polym. Test..

[8] Cole K., Hechler J., Noel D. (1991). A new approach to modeling the cure kinetics of epoxy/amine thermosetting resins. 2. Application to a typical system based on bis [4-(diglycidylamino) phenyl] methane and bis (4-aminophenyl) sulfone. Macromolecules.

[9] Bernath A., Kärger L., Henning F. (2016). Accurate cure modeling for isothermal processing of fast curing epoxy resins. Polymers.

[10] Möllers H., Schmidt C., Meiners D. (2025). Modelling the curing kinetics of DGEBA-MTHPA with DMP-accelerator concentration dependence. Polym. Test..

[11] Tao Q., Pinter G., Antretter T., Krivec T., Fuchs P. (2018). Model free kinetics coupled with finite element method for curing simulation of thermosetting epoxy resins. J. Appl. Polym. Sci..

[12] Hui X., Xu Y., Zhang W., Zhang W. (2022). Cure process evaluation of CFRP composites via neural network: From cure kinetics to thermochemical coupling. Compos Struct..

[13] Wu C., Zhang R., Zhao P., Li L., Zhang D. (2024). Curing simulation and data-driven curing curve prediction of thermoset composites. Sci. Rep..

[14] Wu X., Wei L., Zhang M., Liu Z., Xiao B., Yang X., Yang Z. (2025). Modeling and comparative study on cure kinetics for CFRP: Autocatalytic vs. neural network vs. angle information-enhanced RBF models. Polymers.

[15] Kim G.Y., Melkote S.N., Colton J.S. (2026). Ensemble cure kinetics network (ECK-Net): A method to derive cure kinetics of thermosetting resin. Compos Part A Appl. Sci. Manuf..

[16] Matheron G. (1973). The intrinsic random functions and their applications. Adv. Appl. Probab..

[17] Trochu F. (1993). A contouring program based on dual kriging interpolation. Eng. Comput.

[18] Rasmussen C.E., Williams C.K.I. (2006). Gaussian Processes for Machine Learning.

[19] Wang Q., Gao L., Wang X., Dong Q., Wan G., Du T., Guo Y., Zhang C., Jia Y. (2017). Numerical analysis and fiber Bragg grating monitoring of thermocuring processes of carbon fiber/epoxy laminates. Polym. Test..

[20] Kang J., Sun L., Wang J., Li X., Yang P. (2019). Three-dimensional finite element simulation of temperature and strain in epoxy resin used to electronic packaging during curing. Acta Mater. Compos Sin..

[21] Chern B.C., Moon T.J., Howell J.R., Tan W. (2002). New experimental data for enthalpy of reaction and temperature- and degree-of-cure-dependent specific heat and thermal conductivity of the Hercules 3501-6 epoxy system. J. Compos Mater..

[22] Chern B.C., Moon T.J., Howell J.R. (1993). Measurement of the temperature and cure dependence of the thermal conductivity of epoxy resin. Exp. Heat Transf..

[23] Khoun L., Centea T., Hubert P. (2010). Characterization methodology of thermoset resins for the processing of composite materials—Case study: CYCOM 890RTM epoxy resin. J. Compos Mater..

[24] Jørgensen J.K., Mikkelsen L.P. (2023). Isothermal and Dynamic DSC Dataset for an Epoxy Resin System for Wind Turbine Blade Applications.

[25] Jørgensen J.K., Mikkelsen L.P., Andersen T.L. (2023). Cure characterisation and prediction of thermosetting epoxy for wind turbine blade manufacturing. IOP Conf. Ser. Mater. Sci. Eng..

